# Self-Inhibitory Peptides Targeting the Neisseria gonorrhoeae MtrCDE Efflux Pump Increase Antibiotic Susceptibility

**DOI:** 10.1128/AAC.01542-21

**Published:** 2022-01-18

**Authors:** Benjamin J. Evert, Valentin A. Slesarenko, Jilsy M. J. Punnasseril, Taha, Jian Zhan, Yaoqi Zhou, Evgeny A. Semchenko, Kate L. Seib

**Affiliations:** a Institute for Glycomics, Griffith University, Gold Coast, QLD, Australia

**Keywords:** gonorrhoea, antimicrobial resistance, MtrCDE efflux pump, peptide, antibiotic

## Abstract

Neisseria gonorrhoeae is an increasing public health threat due to its rapidly rising incidence and antibiotic resistance. There are an estimated 106 million cases per year worldwide, there is no vaccine available to prevent infection, and N. gonorrhoeae strains that are resistant to all antibiotics routinely used to treat the infection have emerged. In many strains, antibiotic resistance is mediated by overexpression of the MtrCDE efflux pump, which enables the bacteria to transport toxic antibiotics out of the cell. Genetic mutations that inactivate MtrCDE have previously been shown to render resistant strains susceptible to certain antibiotics. Here, we show that peptides rationally designed to target and disrupt the activity of each of the three protein components of MtrCDE were able to increase the susceptibility of N. gonorrhoeae strains to antibiotics in a dose-dependent manner and with no toxicity to human cells. Cotreatment of bacteria with subinhibitory concentrations of the peptide led to 2- to 64-fold increases in susceptibility to erythromycin, azithromycin, ciprofloxacin, and/or ceftriaxone in N. gonorrhoeae strains FA1090, WHO K, WHO P, and WHO X. The cotreatment experiments with peptides P-MtrC1 and P-MtrE1 resulted in increased susceptibilities of WHO P and WHO X to azithromycin, ciprofloxacin, and ceftriaxone that were of the same magnitude seen in MtrCDE mutants. P-MtrE1 was able to change the azithromycin resistance profile of WHO P from resistant to susceptible. Data presented here demonstrate that these peptides may be developed for use as a dual treatment with existing antibiotics to treat multidrug-resistant gonococcal infections.

## INTRODUCTION

The sexually transmitted infection gonorrhoea is a major public health burden, with an estimated 106 million cases each year (1) and the continuing emergence of antibiotic-resistant Neisseria gonorrhoeae strains (2, 3). Ceftriaxone or ceftriaxone plus azithromycin is currently recommended for treatment in most settings. However, high-level resistance and treatment failure have been reported (4), highlighting the need for new therapeutics. Antibiotic resistance in N. gonorrhoeae is often a result of increased antibiotic efflux from the cell due to the overexpression of the MtrCDE efflux pump (5, 6). The MtrCDE efflux pump plays a role in several aspects of gonococcal infection, including survival in the presence of fatty acids, cationic antimicrobial peptides (7), and human neutrophils (8), as well as in the female mouse genital tract model of gonococcal infection (9).

MtrCDE is made up of three proteins, MtrD (inner membrane transport protein), MtrC (periplasmic membrane fusion protein linking MtrD and MtrE), and MtrE (outer membrane channel protein). The MtrCDE efflux pump can export various classes of antimicrobials, including macrolides (e.g., erythromycin, azithromycin), β-lactam antimicrobials (e.g., penicillin, ceftriaxone), the fluoroquinolone ciprofloxacin, tetracycline, and host-derived antimicrobials (e.g., cationic antimicrobial peptides, long-chain fatty acids) (reviewed in reference 2). Genetically inactivating the MtrCDE pump in antibiotic-resistant strains that overexpress MtrCDE can decrease the organism’s resistance to penicillin, azithromycin, ceftriaxone, and ciprofloxacin by 2- to 16-fold (6, 10). Similarly, transcriptional dampening of the expression of *mtrCDE* can decrease resistance to penicillin and ceftriaxone by 2- to 8-fold *in vitro* and enhance the efficacies of these antibiotics in clearing gonococcal infections in the mouse model (11). Bacterial efflux pumps have been targeted for the development of novel antimicrobials but have not yet entered clinics due to toxicity issues and a lack of methods to guide rational drug design (reviewed in reference 12).

A potential new option for antimicrobials for antibiotic-resistant bacteria is self-inhibitory peptides, which are peptides that are designed from the sequence of the protein that they target. We recently showed that the self-derived peptide KFF-EcH3 {comprised of 20 amino acids derived from the Escherichia coli essential protein methionine aminopeptidase (MetAP) fused to a cell-penetrating peptide [(KFF)_3_K] and a flexible linker (GSG)} can disrupt the secondary and tertiary structures of MetAP, resulting in species-specific inhibition of cell growth, with no development of resistance seen (13). Similarly, the N. gonorrhoeae MetAP self-derived peptide KFF-NgH1 can inhibit gonococcal growth and gonococcal infection of a human cervical epithelial cell model (13). Here, we describe self-derived peptides that are rationally designed based on the same strategy to target the MtrC, MtrD, and MtrE proteins of the MtrCDE efflux pump and characterize their ability to increase susceptibility in N. gonorrhoeae to antibiotics previously and currently recommended for treatment.

## RESULTS AND DISCUSSION

### Rational design of MtrCDE-inhibitory peptides.

**FIG 1:**
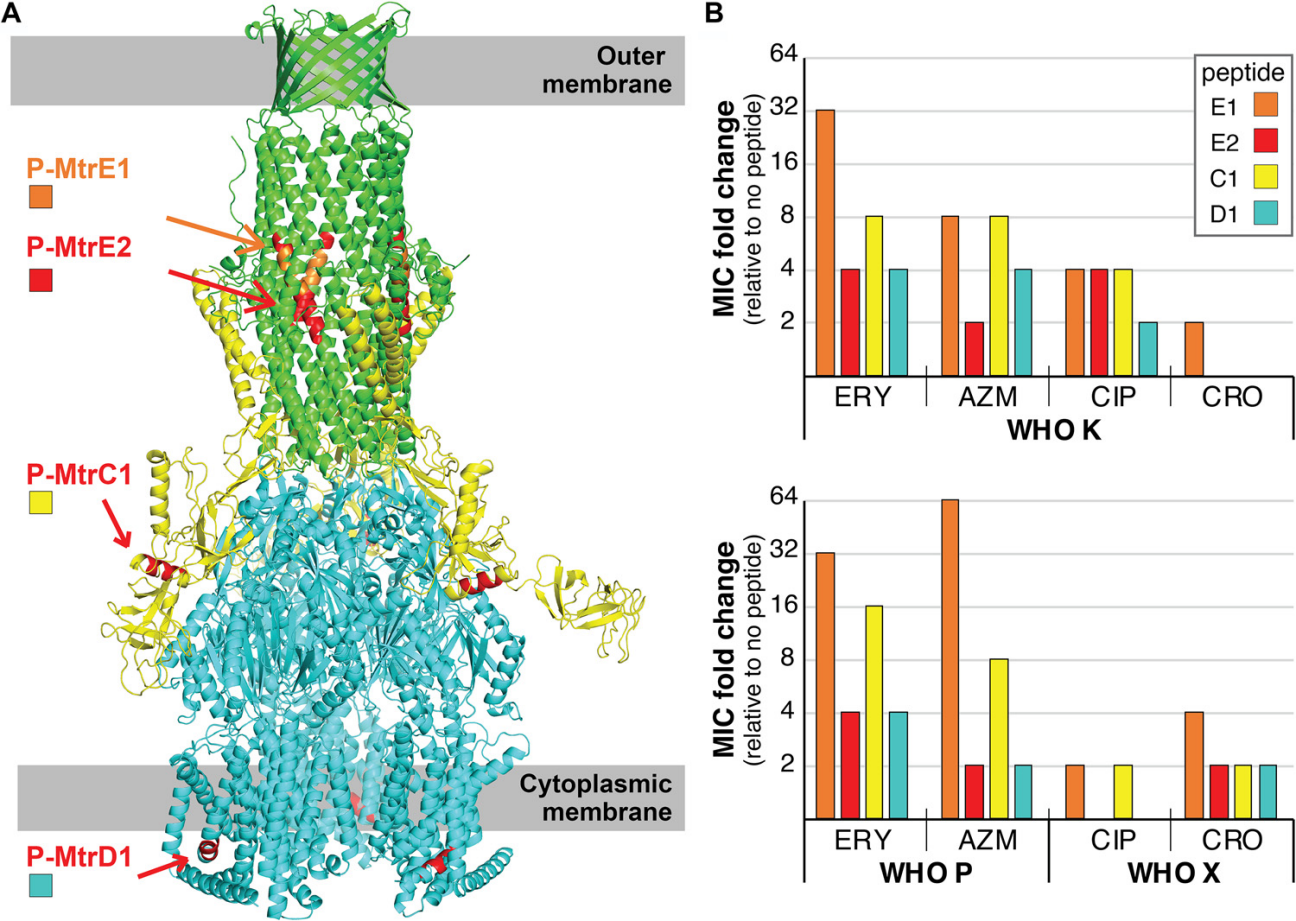
Representation of MtrCDE regions targeted by self-derived peptides and their impact on antibiotic susceptibility. (A) The complex structure of the Neisseria gonorrhoeae MtrCDE multidrug efflux pump was modeled using mtss1Dock (27) to dock the structures of MtrD (cyan; PDB accession no. 4MT1) (28), MtrE (green; PDB accession no. 4MT0) (29), and MtrC (yellow; modeled by Sparks-X [30]) together. The alpha-helical segments of the MtrE, MtrC, and MtrD proteins corresponding to the self-derived peptides are labeled in orange (P-MtrE1) or red (P-MtrE2, P-MtrC1, P-MtrD1). (B) The fold decreases in the MICs of erythromycin (ERY), azithromycin (AZM), ciprofloxacin (CIP), and ceftriaxone (CRO) for the WHO K, P, and X strains in the presence of self-derived peptides, relative to no peptide, are shown. MIC values are shown in Table 2.

Self-derived peptides targeting alpha-helical segments of each of the three proteins of the MtrCDE pump were selected from the amino acid sequence of the proteins based on their intraprotein DFIRE (distance-scaled, finite, ideal-gas reference state) interaction energy score (13) (Table 1; Fig. 1). The lower the DFIRE score, the stronger the predicted interaction energy between a helical peptide segment and the remaining portion of the protein and, therefore, the higher the probability that the self-derived peptide with the same sequence as that portion of the protein can disrupt the protein structure and inhibit function by breaking into the folding core of the protein to replace the original helix during the dynamic opening motion of the protein.

**TABLE 1:** MtrCDE self-inhibiting peptides

Peptide^*a*^	Sequence^*b*^	Peptide length	Energy^*c*^
P-MtrE1	*(KFF)_3_KGSG* **HLSLIATV**	8	–3.74
P-MtrE1a	**HLSLIATV**	8	–3.74
P-MtrE2	*(KFF)_3_KGSG*DAA**HLSLIATV**AKAYFNERY	20	−2.95
P-MtrC1	*(KFF)_3_KGSG*VMKLRRQI	8	−2.09
P-MtrD1	(*KFF)_3_KGSG*VFLVPLFY	8	−6.69

a Peptides are named after the MtrCDE component that they target. Peptides were solubilized in water (P-MtrC1, P-MtrE1, and P-MtrE1a) or DMSO (P-MtrD1 and P-MtrE2). Peptides were synthesized by GenScript (NJ, USA) at >90% purity, with N-terminal acetylation and C-terminal amidation.

b The peptide sequence includes the 13-amino-acid N-terminal cell-permeating peptide and GSG flexible linker [(KFF)_3_KGSG; shown in italics when present] and the 8 (in bold)- or 20-amino-acid MtrCDE-derived peptide.

c The intraprotein DFIRE interaction energy score was calculated between the peptide segment and the remaining portion of the protein.

Four peptides with low DFIRE scores were selected for further investigation, one targeting each of the proteins MtrC and MtrD and two targeting MtrE. To facilitate entry of the peptides into the bacterial cell, they were synthesized with the (KFF)_3_K cell-permeating peptide, which is known to penetrate bacterial cells without toxicity (13, 14), and a flexible Gly-Ser-Gly (GSG) linker on the N terminus (named P-MtrE1, P-MtrE2, P-MtrC1, and P-MtrD1 for simplicity [“P” stands for “peptide”]). Two control peptides were also synthesized to include the (KFF)_3_KGSG linker alone and P-MtrE1a, which has a sequence identical to that of P-MtrE1 but without the cell-permeating peptide.

### Self-inhibitory peptides targeting MtrCDE increase gonococcal antibiotic susceptibility.

Six peptides were tested against N. gonorrhoeae strains with various antimicrobial resistance phenotypes and with either standard MtrCDE expression (strain FA1090) or MtrCDE overexpression (strains WHO K, WHO P, and WHO X [15, 16]) (Table 2). The MIC of each peptide was initially determined according to Clinical and Laboratory Standards Institute (CLSI) guidelines (i.e., the lowest concentration of an antimicrobial agent that prevents visible growth of a microorganism in a broth dilution assay over 20 h of growth) (17). Peptide MICs were ≥25 μg/ml for strain FA1090 and ≥100 μg/ml for WHO strains. Subsequent experiments were conducted with subinhibitory peptide concentrations (20 μg/ml for FA1090; 50 μg/ml for WHO K, WHO P, and WHO X).

**TABLE 2:** MICs of selected antibiotics in the absence and presence of self-derived peptides targeting each component of the MtrCDE efflux pump

Strain (MtrCDE expression^*c*^)	MIC (μg/ml)^*a*^			
	ERY (ND)	AZM (S ≤ 0.25, R > 0.5)	CIP (S ≤ 0.03, R > 0.06)	CRO (S ≤ 0.125, R > 0.125)
FA1090 (standard)	0.2	0.063 (S)	0.01 (S)	<0.0078 (S)
+ P-MtrE1	0.1	0.008	—	—
+ P-MtrE1a	0.2	0.063	—	—
+ P-MtrE2	0.1	0.063	—	—
+ P-MtrC1	0.05	0.031	—	—
+ P-MtrD1	0.1	0.031	—	—
(KFF)_3_KGSG	0.2	0.063	—	—
WHO K (overexpressed)	2	0.25 (S)	64 (HLR)	0.031 (S)
+ P-MtrE1	0.063	0.031	16	0.016
+ P-MtrE1a	2	0.25	64	0.031
+ P-MtrE2	0.5	0.125	16	0.031
+ P-MtrC1	0.25	0.031	16	0.031
+ P-MtrD1	0.5	0.063	32	0.031
(KFF)_3_KGSG	2	0.25	64	0.031
WHO P (overexpressed)	16	4 (R)	0.004 (S)	0.004 (S)
+ P-MtrE1	0.25	0.063* (S)	—	—
+ P-MtrE1a	16	4	—	—
+ P-MtrE2	4	2	—	—
+ P-MtrC1	1	0.5 (I)	—	—
+ P-MtrD1	4	2	—	—
(KFF)_3_KGSG	16	4	—	—
WHO X (overexpressed)	2	0.5 (I)	64 (HLR)	2 (HLR)
+ P-MtrE1	—	—	32^*b*^	0.5^*b*^
+ P-MtrE1a	—	—	64	2
+ P-MtrE2	—	—	64	1
+ P-MtrC1	—	—	32^*b*^	1
+ P-MtrD1	—	—	64	1
(KFF)_3_KGSG	—	—	64	2

a ERY, erythromycin; AZM, azithromycin; CIP, ciprofloxacin; CRO, ceftriaxone. MICs were determined using the microdilution method per CLSI guidelines (17) in the absence or presence of 20 μg/ml peptide (FA1090), 50 μg/ml peptide (WHO K, WHO P, WHO X), or the (KFF)_3_KGSG cell-permeating peptide and linker only. Antimicrobial resistance phenotypes are indicated (S, susceptible; I, intermediate susceptible; R, resistant; HLR, high-level resistant; ND, not determined [16]) based on EUCAST breakpoints (www.eucast.org), as indicated. WHO reference strain antibiotic MICs determined for this study were equivalent to published MICs (16), except for the erythromycin MIC for WHO, which was above the reported MIC, likely due to the different methods used to determine MIC. —, not tested.

b Peptide reduced the MIC by the same magnitude (fold) as that seen between the wild-type and MtrCDE knockout strains (6).

c MtrCDE expression as reported previously (5, 15), with overexpression due to either deletion of an A (WHO K, X) or an A-to-C (WHO P) polymorphism in the promoter of the MtrCDE efflux pump (MtrR).

The MICs of erythromycin, azithromycin, ciprofloxacin, and ceftriaxone were then determined in the absence and presence of each peptide. Cotreatment with the peptides P-MtrE1, P-MtrE2, P-MtrC1, and P-MtrD1 reduced the MICs of the four antibiotics tested by at least 2-fold for at least one N. gonorrhoeae strain (Table 2; Fig. 1B). P-MtrE1 had the strongest inhibitory effect, with a 2- to 64-fold reduction in antibiotic MICs for all strains tested (Table 2; Fig. 1B). P-MtrE1 reduced the WHO X ciprofloxacin and ceftriaxone MICs by the same magnitude as that of the MIC reduction previously seen between the MtrCDE knockout and wild-type strains (6). This indicates that the MtrCDE efflux pump was inactivated by the peptide. P-MtrE1 and P-MtrC1 also reduced the WHO P azithromycin MIC by the same magnitude as that for the MtrCDE knockout strain versus wild-type strains, changing the resistance profile of this strain from azithromycin resistant to susceptible (P-MtrE1) or intermediate susceptible (P-MtrC1). Antibiotic resistance is multifactorial in many gonococcal strains (16), which may explain why the MtrCDE-targeting peptides were not able to render all strains sensitive to all antibiotics. For example, in addition to overexpressing MtrCDE, WHO X has a mosaic *penA* allele that is the main resistance determinant for extended-spectrum cephalosporins, such as ceftriaxone (18). Ciprofloxacin resistance in WHO K and WHO X is also driven by mutations in GyrA and ParC (15, 16).

### Inhibitory peptide activity against MtrE is dependent on cell membrane permeation, peptide size, and concentration.

Analyses of the peptides targeting MtrE were conducted to further investigate their ability to increase gonococcal antibiotic susceptibility. P-MtrE1a, which has an amino acid sequence identical to that of P-MtrE1 without the (KFF)_3_KGSG sequence, is not able to increase susceptibility to any antibiotic for any strain against which P-MtrE1 had an effect (Table 2), indicating that the cell-permeating peptide sequence is necessary for entry of the peptide into the bacterial cell and disruption of the MtrCDE pump. The (KFF)_3_KGSG cell-permeating peptide sequence alone does not affect the growth or survival of N. gonorrhoeae (13) and does not affect N. gonorrhoeae’s susceptibility to the antibiotics tested (Table 2).

Two peptides of different lengths targeting the same alpha-helical region of MtrE were tested to investigate the impact of peptide length on their activity. P-MtrE1 contains 8 amino acids of MtrE and mediated a 2- to 8-fold-higher reduction in MIC than P-MtrE2, which is a 20-amino-acid peptide that contains the 8-amino-acid P-MtrE1 sequence within it (Fig. 1B). In this instance, this suggests that a shorter peptide is more effective at disrupting the target protein’s activity. This is consistent with P-MtrE1 having a lower DFIRE score than P-MtrE2, which predicts a stronger interaction between the P-MtrE1 helical peptide segment and the remaining portion of the protein. Also, longer peptides may not be able to target the structural regions as effectively due to steric hindrance.

**FIG 2:**
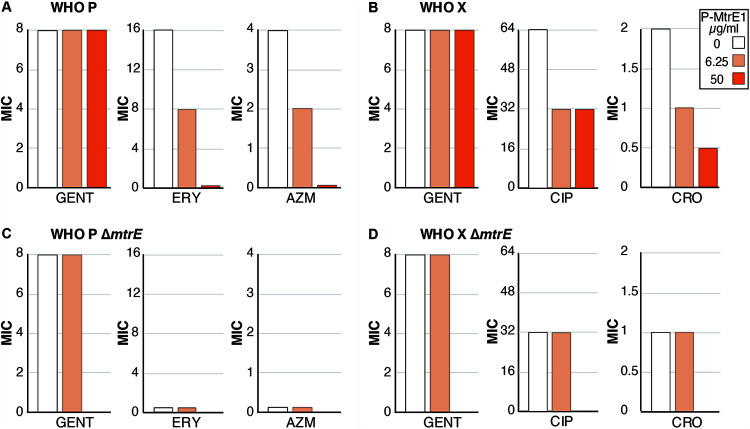
Impact of the P-MtrE1 self-derived peptide on the antibiotic MICs for wild-type and Δ*mtrE* knockout strains of WHO P and WHO X. (A) MICs of gentamicin (GENT), erythromycin (ERY), and azithromycin (AZM) for N. gonorrhoeae strain WHO P; (B) MICs of gentamicin, ciprofloxacin (CIP), and ceftriaxone (CRO) for N. gonorrhoeae strain WHO X in the presence of 0, 6.5, or 50 μg/ml of self-derived peptide P-MtrE1; (C) MICs of gentamicin, erythromycin, and azithromycin for N. gonorrhoeae strain WHO P Δ*mtrE*; (D) MICs of gentamicin, ciprofloxacin, and ceftriaxone for N. gonorrhoeae strain WHO X Δ*mtrE* in the presence of 0 or 6.5 μg/ml of P-MtrE1.

The impact of P-MtrE1 on gonococcal antibiotic susceptibility was concentration dependent for erythromycin, azithromycin, and ceftriaxone when tested against WHO P and WHO X (Fig. 2A and B). Concentrations as low as 6.25 μg/ml of peptide are able to reduce the MICs of erythromycin, azithromycin, ciprofloxacin, and ceftriaxone (Fig. 2A and B). A direct comparison between the wild-type and a *ΔmtrE* mutant strain was not possible due to the intrinsic sensitivity of the *ΔmtrE* knockout to antimicrobial compounds (6). However, there was no change in the antibiotic susceptibility of the WHO P Δ*mtrE* strain to erythromycin or azithromycin or of the WHO X Δ*mtrE* strain to ciprofloxacin or ceftriaxone in the presence or absence of subinhibitory P-MtrE1 peptide concentrations, whereas a change in antibiotic susceptibility was observed for the wild-type strain under the same conditions (Fig. 2C and D). MtrE1 also had no impact on the susceptibilities of the WHO P and WHO X wild-type and Δ*mtrE* strains to gentamicin (Fig. 2A to D), which is an antibiotic that is not subject to efflux by MtrCDE (19).

It is likely that the peptides targeting the proteins of the MtrCDE pump disrupt the secondary and tertiary structures of the proteins, as seen previously for similar self-derived peptides that target MetAP of E. coli and N. gonorrhoeae (13). The peptides are predicted to act by breaking into the folding core of the protein to replace the original helix during the dynamic opening motion of the protein (13). This may prevent correct assembly and/or cause disassembly of the individual proteins and the tripartite pump. Studies have identified functionally important residues of MtrD (19) and MtrE (20); however, the P-MtrD1 peptide and the P-MtrE1 and P-MtrE2 peptides do not overlap these residues and do not target the same region of the protein based on available crystal structures (MtrD PDB accession no. 4MT1, MtrE PDB accession no. 4MT0). As is true for many antibiotics, additional testing is needed to determine their spectra of activity, as bacterial species in the microbiome may also be affected by the MtrCDE-targeting peptides. However, our sequence analysis indicated that bacterial species commonly found in the female genital tract microbiome, including *Lactobacillus*, *Gardnerella*, *Atopobium*, and *Prevotella*, do not contain an MtrCDE homologue. Since the peptides investigated are relatively short, they may have some identity to segments of other bacterial proteins. However, the likelihood of off-target effects is reduced by the mode of action of the self-derived peptides being linked to protein structure, as well as sequence.

### Self-derived MtrCDE peptides are not toxic to human cells.

**FIG 3:**
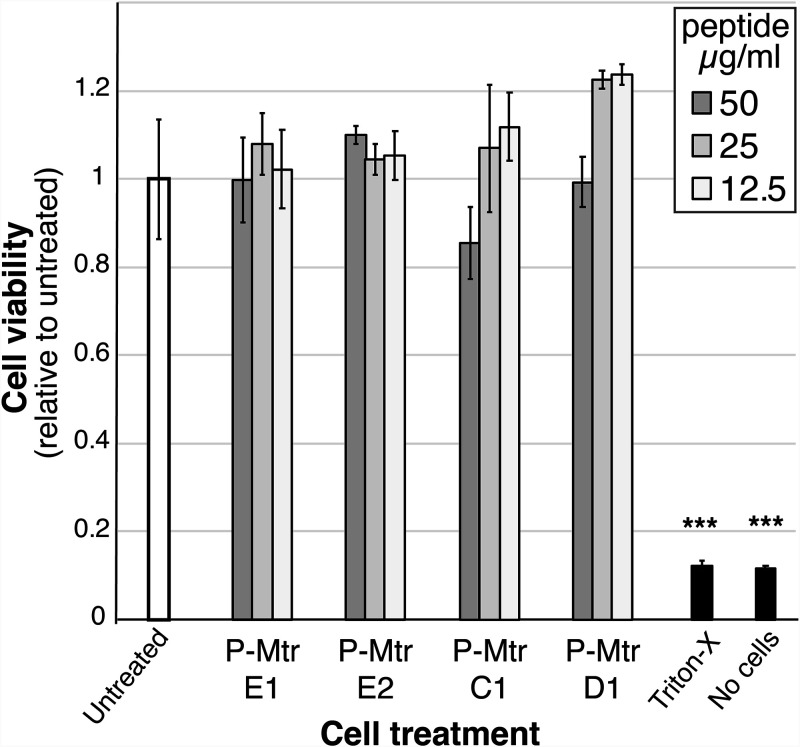
ME180 cell viability in the presence of MtrCDE self-derived peptides. Cell viability (relative to that of untreated, negative-control cells) is shown for human cervical ME180 cells according to the CellTiter 96 AQ_ueous_ one-solution cell proliferation assay. ME180 cells were grown to confluence in a 96-well plate, washed, and then incubated with 12.5 to 50 μg/ml peptide for 2 h before addition of the detection reagent. The cells were then incubated a further 2 h before measurement of absorbance at 490 nm. Controls used contained either live cells with medium only (Untreated), cells treated with 0.1% (vol/vol) Triton X-100 (dead cells), or medium with no cells (No cells). The graph shows the average absorbance from three independent replicates ±1 standard deviation. The reduced cell viability of the Triton X-100-treated cells relative to that of untreated cells is indicated (***, *P* value ≤ 0.001 by a two-tailed Student *t* test). No significant reduction in cell viability was seen for cells treated with peptides relative to that of untreated cells (*P* value ≥ 0.09).

To determine if the peptides display toxicity to human cells, metabolic activity was evaluated for ME180 human cervical cells incubated with 12.5 to 50 μg/ml peptide for 2 h, using a cell proliferation assay. There was no statistically significant difference in ME180 cell viability between the untreated and the treated cells, indicating that none of the peptides were cytotoxic at concentrations up to 50 μg/ml (Fig. 3). Light microscopy was also used to confirm that there was no visible damage to the cells in the presence of the peptides, with ME180 having similar morphologies in the presence and absence of peptides.

### Summary.

We have shown that self-derived peptides selected from alpha-helical structural regions of the MtrC, MtrD, and MtrE proteins can be used to inhibit the MtrCDE efflux pump and increase susceptibility to antibiotics in a concentration-dependent manner. We also showed that none of the peptides displayed toxicity to ME180 human cells at concentrations necessary to inhibit the MtrCDE pump. The WHO Global Health Sector Strategy on sexually transmitted infections (STIs) has set targets for reducing global gonorrhoea incidence by 90% by 2030; optimizing medicines and treatment regimens and developing new, more effective medicines for N. gonorrhoeae are included as priorities to achieving this goal (21). The self-derived MtrCDE peptides characterized here may be further developed for use as a novel dual therapy in conjunction with existing antibiotics to treat antibiotic-resistant N. gonorrhoeae infections.

## MATERIALS AND METHODS

### Strains and growth conditions.

N. gonorrhoeae strains used in this study include FA1090 (standard MtrCDE expression) (22), WHO K (MtrCDE overexpression) (15), WHO P (MtrCDE overexpression) (15), and WHO X (MtrCDE overexpression) (16). Wild-type strains were grown on GC agar or in GC broth (Oxoid) supplemented with IsoVitaleX (Bacto) at 37°C in 5% CO_2_. *mtrE* knockout strains of N. gonorrhoeae WHO X and WHO P (Δ*mtrE*) were generated by transforming bacteria with 1 μg of a linear DNA fragment (IDT) containing aminoglycoside 3′-phosphotransferase gene (NCBI accession no. VTT49712.1) flanked by 500-bp terminal fragments of the N. gonorrhoeae FA1090 *mtrE* gene (GenBank accession no. AE004969.1, positions 1 to 500 and 903 to 1403), as well as the gonococcal DNA uptake sequence (ATGCCGTCTGAA) at the 5′ end (23). E. coli strain DH5α, used for cloning, was grown using Luria-Bertani agar or broth (Oxoid) at 37°C. The media were supplemented with 100 μg/ml ampicillin or 50 μg/ml kanamycin, as required.

### Peptides.

Self-inhibitory peptides targeting alpha-helical segments of the three proteins of the MtrCDE pump were selected based on their intraprotein DFIRE interaction energy score, as predicted by computational analysis using DFIRE (24) as previously described (13). Peptides used in this study (Table 1) were synthesized by GenScript (NJ, USA) at >90% purity with N-terminal acetylation and C-terminal amidation and are shown in Table 2. Peptides were resuspended in water (P-MtrC1, P-MtrE1, and P-MtrE1a) or dimethyl sulfoxide (DMSO) (P-MtrD1 and P-MtrE2) and used at concentrations up to 20 μg/ml for FA1090 and 50 μg/ml for WHO strains.

### Antibiotic and peptide susceptibility testing.

The MIC was determined according to the CLSI guidelines (i.e., the lowest concentration of an antimicrobial agent that prevents visible growth of a microorganism in a broth dilution assay over 20 h) (17). Experiments were performed using a microdilution method in triplicate wells. Control wells contained medium with 1% water or DMSO with and without bacteria, which had no impact on bacterial survival or growth.

High-level resistance, resistance, intermediate resistance, and susceptibility phenotypes were determined per previously determined MIC values (15) using the interpretive criteria published by the Swedish Reference Group on Antibiotics (SGRA) (25). For cotreatment assays (peptide plus antibiotic), the antibiotic dilutions were made up in GC broth that contained the peptide at the appropriate concentration. The experiments were performed with peptide-only and antibiotic-only controls. Each well was inoculated with 10 μl of the appropriate N. gonorrhoeae strain at an optical density (absorbance at 600 nm) of 0.1 (∼1 × 10^6^ CFU/ml). All experiments were performed with triplicate samples on at least three occasions.

### Cytotoxicity assays.

Cell toxicity assays were performed using the CellTiter 96 AQ_ueous_ one-solution cell proliferation assay (Promega) per the manufacturer’s instructions. The assays were performed using ME180 cells grown to confluence in a 96-well plate in DMEM (Gibco) supplemented with 10% (vol/vol) fetal bovine serum (FBS) (Gibco) at 37°C and 5% CO_2_, as previously described (26). Cell monolayers were washed with Hanks’ balanced salt solution (HBSS) (Gibco) and then incubated with 12.5 to 50 μg/ml peptide for 2 h before addition of the reagent (triplicate wells). Cells were then incubated a further 2 h before absorbance was measured at 490 nm. Control wells contained either medium with no cells, medium without peptide (live cells), or medium with 0.1% (vol/vol) Triton X-100 (dead cells). Cell viability was calculated relative to that of a no-treatment control (live cells). Statistics were performed using one-way analysis of variance (ANOVA) and Student's *t* test.
